# Supplementary material for: Isolation, Characterization and Pharmacological Investigations of a New Phenolic Compound along with Four Others Firstly Reported Phytochemicals from Glycosmis cyanocarpa (Blume) Spreng
Source: Molecules. 2022 Sep 14;27(18):5972. doi: 10.3390/molecules27185972 (PMC9503977; doi:10.3390/molecules27185972)
Supplement: Supplementary file 1 [file molecules-27-05972-s001.zip › molecules-1909037-supplementary.pdf]

Table S1. ^{1}H NMR Spectroscopic Data (400 MHz, $CDCl_3$) for Compound 1.

		**Compound 1**		**2-methoxy-4-propyl phenol** (Monguchi et al., 2011)	
**Position**	**δ*C**	**δH**	**HMBC**	**δH**	**δC**
1	132.32	---	---	---	134.5
2	111.05	6.53, br s	121.32 (C-6), 143.90 (C-4)	6.63, 1H br s	111.0
3	146.40	---	---		---
4	143.90	---	---		---
5	114.09	6.82, 1H d (*J* = 8.0 Hz)	132.32 (C-1), 146.40 (C-3)	6.81, 1H d (*J* = 8.2 Hz)	114.1
6	121.32	6.60, 1H d (*J* = 8.0 Hz)	143.90 (C-4)	6.63-6.65 2H m	120.9
7	46.47	2.57, 2H br t (*J* = 6.8, Hz)	---	2.50, 2H t (*J* = 7.7 Hz)	---
8	39.23	**2.55, 1H m**	46.47 (C-7)	**1.59, 2H dd (*J* = 7.2, 7.7 Hz)**	37.6
9	73.30	3.93, 2H m		---	---
OCH_3-3	55.79	3.85, 3H s		3.81, 3H s	55.7
CH_3-8	14.14	**0.86, 3H m**	39.23 (C-8)	**0.92, 3H t (*J* = 7.2 Hz)**	13.7

Table S2. ¹H NMR Spectroscopic Data (400 MHz, $CDCl_3$) for Compound 2.

**Position**	**Compound 2**			**3-methyl-1*H*-indole** (Abraham and Reid, 2002)
	**δ^*_C**	**δ_H**	**HSQC**	**δ_H**
2	121.96	7.04, 1H d (J = 2.0 Hz)	121.96	6.96
3	100.61	---	---	---
4	118.73	7.66, 1H d (J = 7.8 Hz)	118.73	7.58
5	119.56	7.13, 1H dd (J = 7.8, 7.2 Hz)	119.56	7.12
6	121.96	7.20, 1H dd (J = 7.8, 7.2 Hz)	121.96	7.19
7	110.20	7.36, 1H d (J = 8.0 Hz)	110.20	7.30
8	139.50	---	---	---
9	136.30	---	---	---
NH	-	7.99, 1Hd (J = 2.0 Hz)	---	---
CH_3- 3	13.06	2.36, 3H s	13.06	2.34

Table S3. ^{1}H NMR Spectroscopic Data (400 MHz, $CDCl_3$) for Compound 3.

Position	Compound 3 3	Tri-*trans*poly-*cis* prenol- 12 (Ibata et al., 1983)
	δ_H	δ_H
H-1	4.09, 2H d (J = 7.2 Hz)	4.09
H-2	5.44, 1H t (J = 7.2Hz)	5.44
H-3	1.74, 3H s	1.74
H-4,5,8,9,12,13, 16,17,20,21,24,25, 28,29, 32,33,36,37,40,41,44,45	2.03, 44H m	2.03, m*
H-6,10,14,18,22,26,30,34,38,42,46	5.12, 11H br s	5.12, br s
CH_3-trans 35,39,43 and ω- CH_3- trans	1.60, 12H s	1.60
CH_3-cis 7,11,15,19,23,27,31 and ω- CH_3-cis	1.68, 24H s	1.68

Table S4. ^{1}H NMR Spectroscopic Data (400 MHz, $CDCl_3$) for Compound 4.

Position	Compound 4	Stigmasterol (Pateh et al., 2009)
	δ_H	δ_H
H-3	3.52, 1H m	3.52, 1H m
H-6	5.35, 1H d (*J* = 4.2 Hz)	5.36, 1H bs
H-18	0.70, 3H s	0.70, 3H s
H-19	1.01, 3H s	1.01, 3H s
H-21	1.02, 3H d (*J* = 7.6 Hz)	1.02, 3H d (*J* = 7.5 Hz)
H-22	5.15, 1H dd (*J* = 15.1, 8.6 Hz)	5.14,1H m
H-23	5.01, 1H dd (*J* = 15.1, 8.6 Hz)	4.98, 1H m
H-26	0.80, 3H d (*J* = 6.8 Hz)	0.80, 3H d (*J* = 6.5 Hz)
H-27	0.85, 3H d (*J* = 6.1 Hz)	0.85, 3H d (*J* = 6.5 Hz)
H-29	0.80, 3H t (*J* = 7.0 Hz)	0.80, 3H t (*J* = 7.5 Hz)

Table S5. ^{1}H NMR Spectroscopic Data (400 MHz, $CDCl_3$) for Compound 5.

**Position**	**Compound 5**	**β-sitosterol** (Pateh et al., 2009)
	**δ_H**	**δ_H**
H-3	3.55, 1H m	3.53, 1H m
H-6	5.37, 1H d (*J* = 4.8 Hz)	5.37, 1H bs
H-18	0.71, 3H s	0.68, 3H s
H-19	1.07, 3H s	1.01, 3H s
H-21	0.94, 3H d (*J* = 7.6 Hz)	0.92, 3H d (*J* = 7.5 Hz)
H-26	0.82, 3H d (*J* = 6.5 Hz)	0.81, 3H d (*J* = 6.5 Hz)
H-27	0.84, 3H d (*J* = 6.5 Hz)	0.83, 3H d (*J* = 6.5 Hz)
H-29	0.86, 3H t (*J* = 7.6 Hz)	0.85, 3H t (*J* = 7.5 Hz)

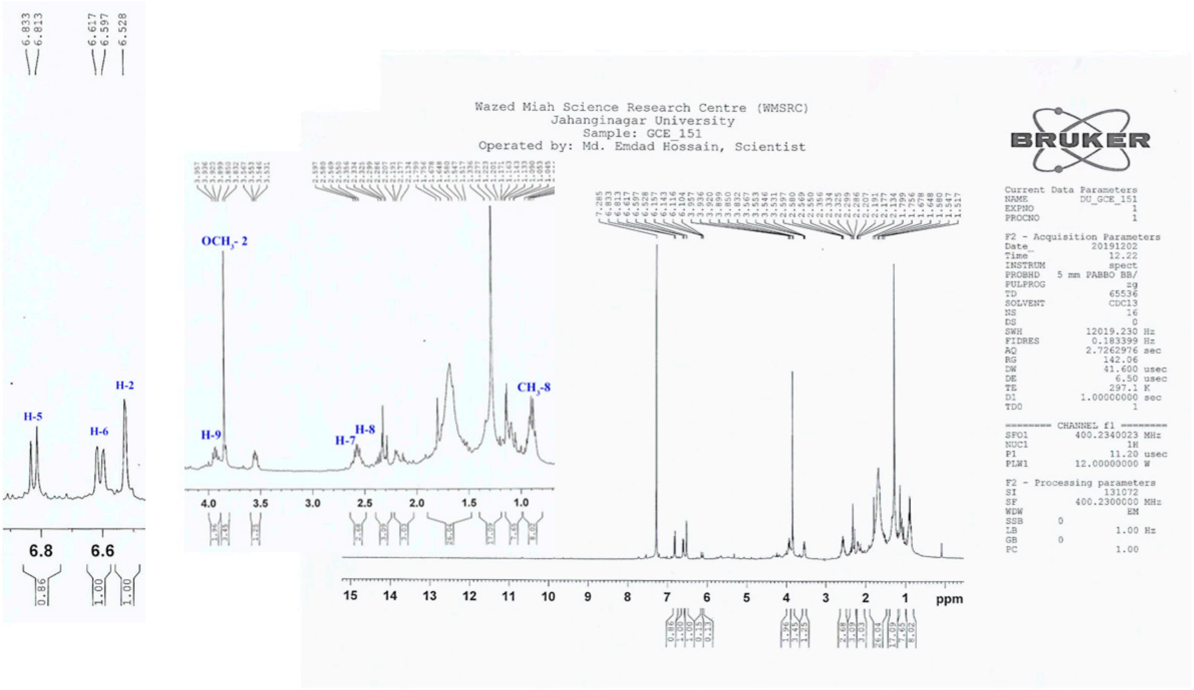

Figure S1. ^{1}H NMR spectrum (400 MHz, $CDCl_3$) of Compound 1.

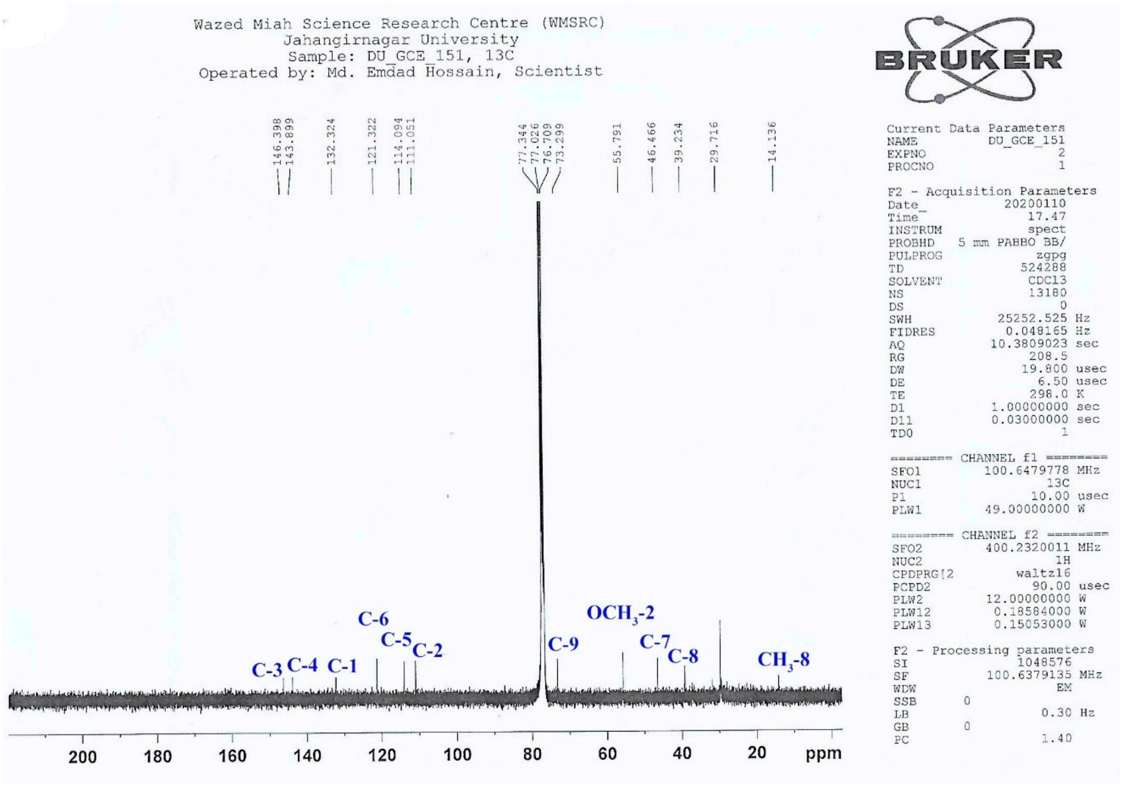

Figure S2. ^{13}C NMR spectrum (400 MHz, $CDCl_3$) of Compound 1.

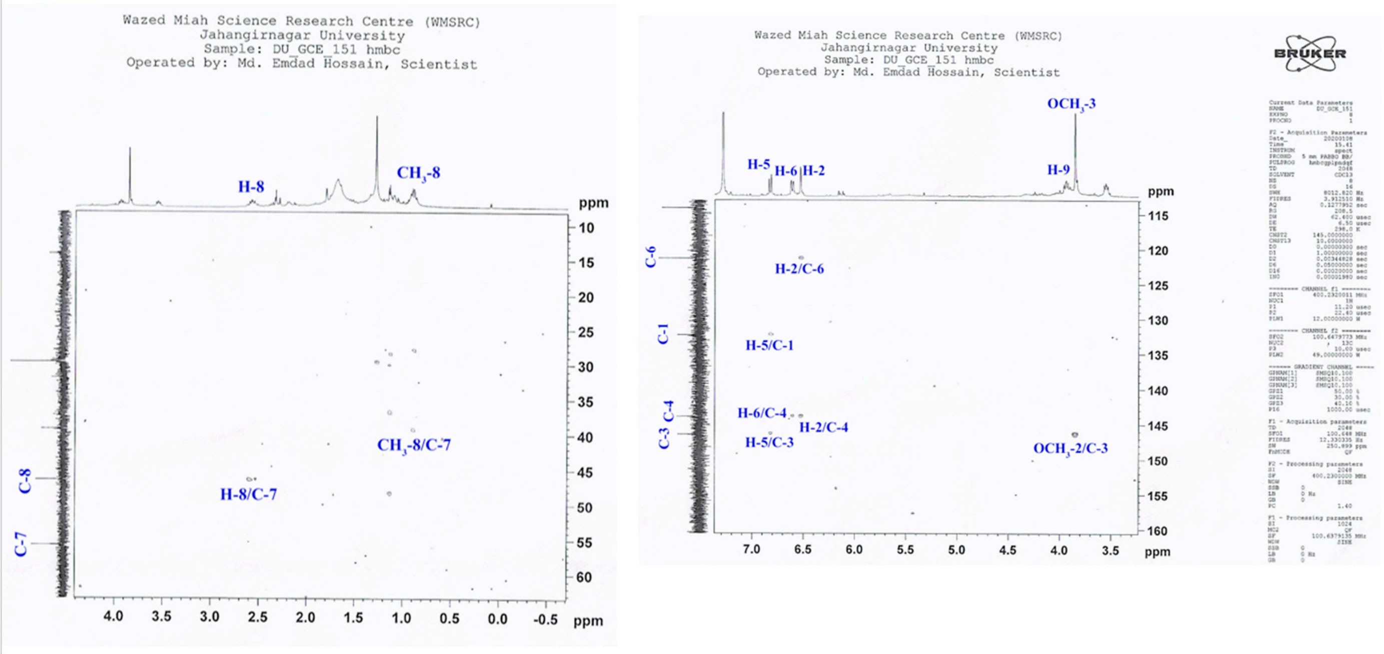

Figure S3. HMBC spectrum (400 MHz, $CDCl_3$) of Compound 1.

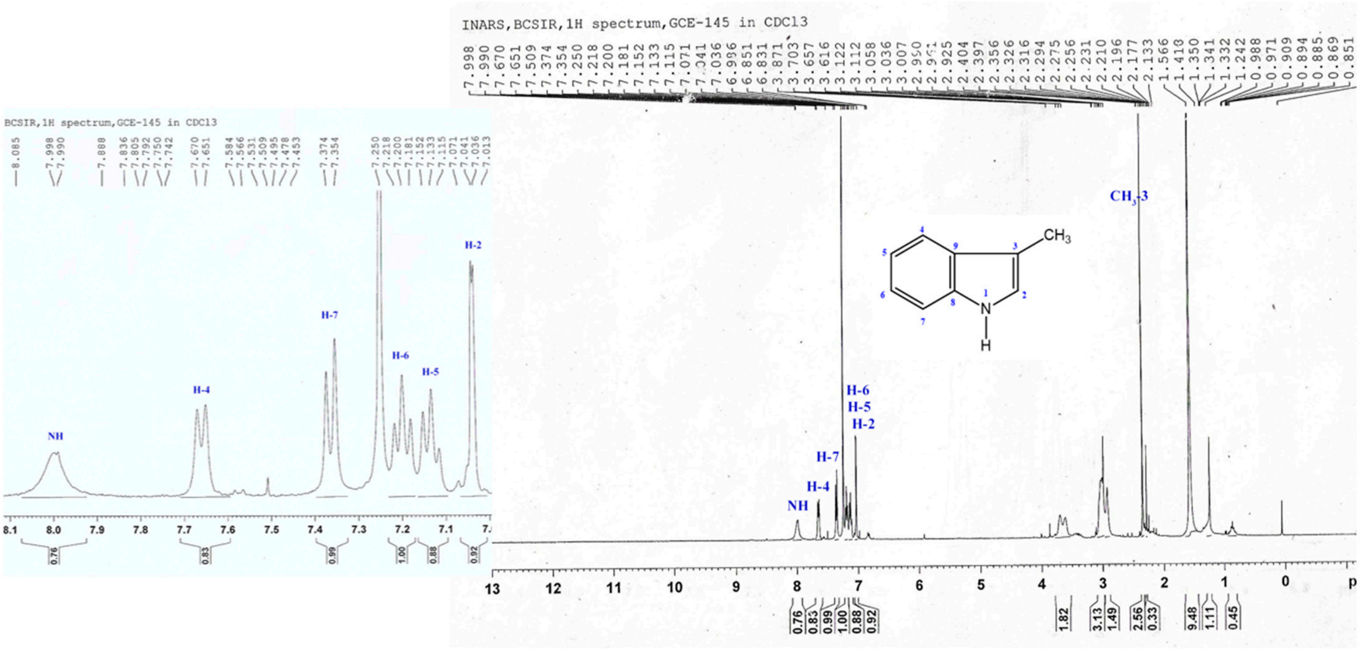

Figure S4. ^{1}H NMR spectrum (400 MHz, $CDCl_3$) of Compound 2.

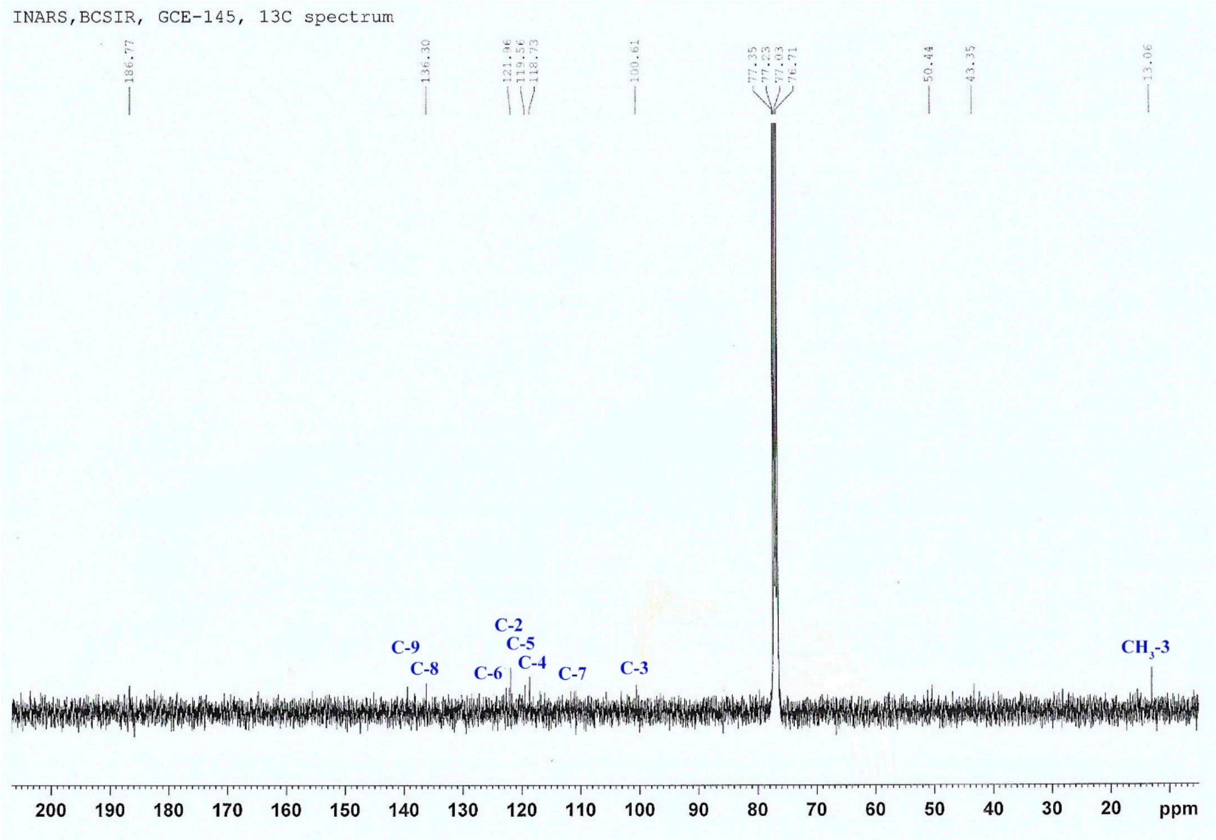

Figure S5. ^{13}C NMR spectrum (400 MHz, $CDCl_3$) of Compound 2.

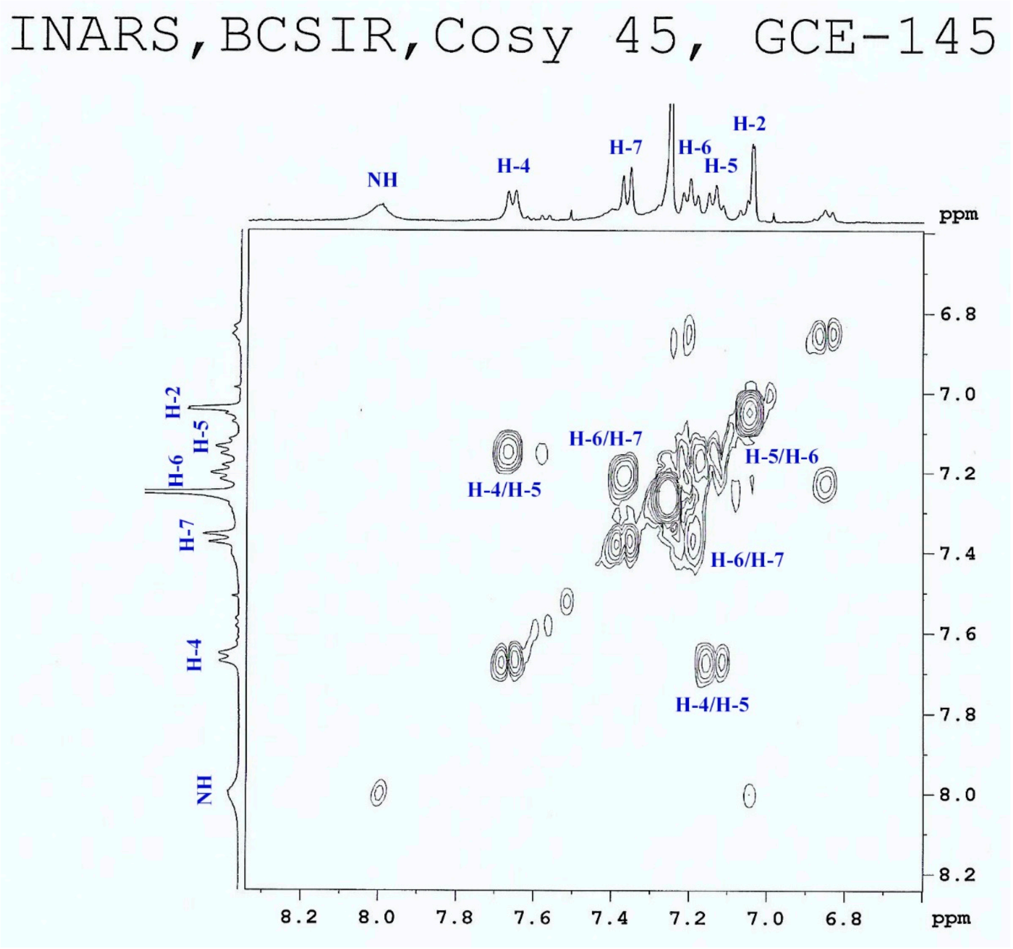

Figure S6. COSY spectrum (400 MHz, $CDCl_3$) of Compound 2.

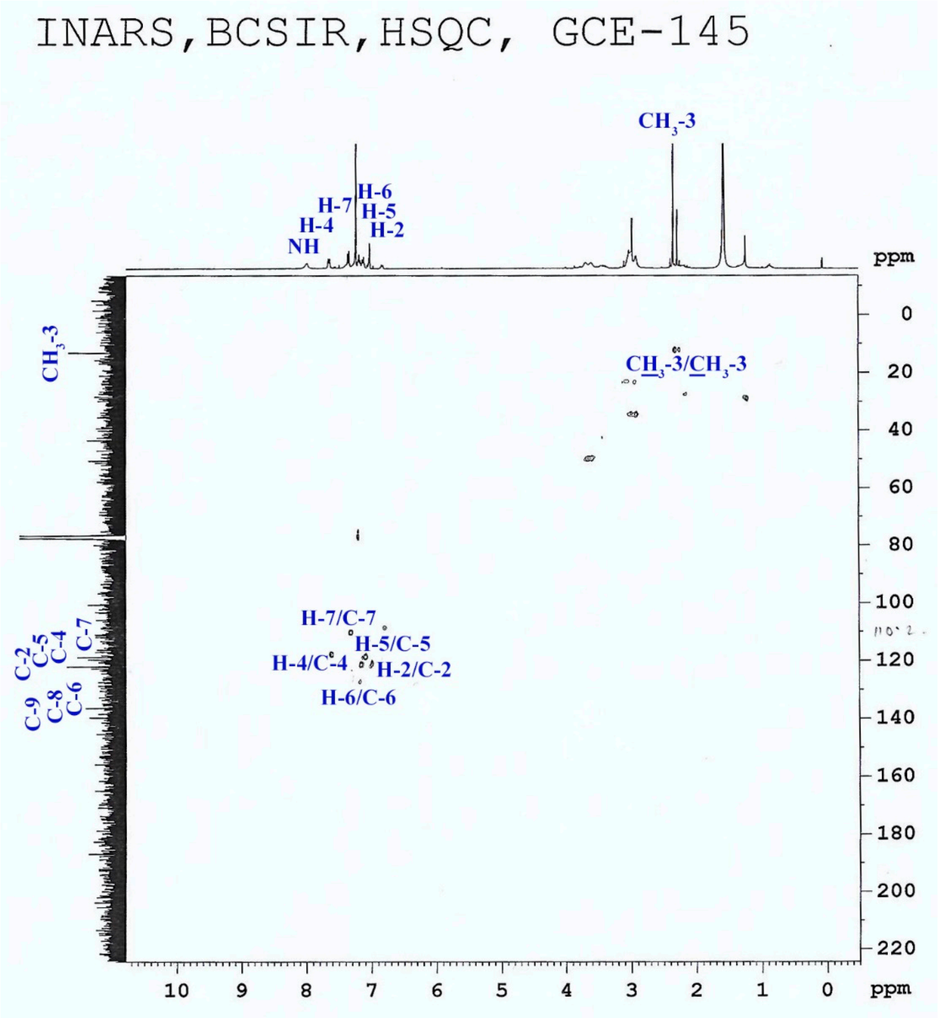

Figure S7. HSQC spectrum (400 MHz, $CDCl_3$) of Compound 2.

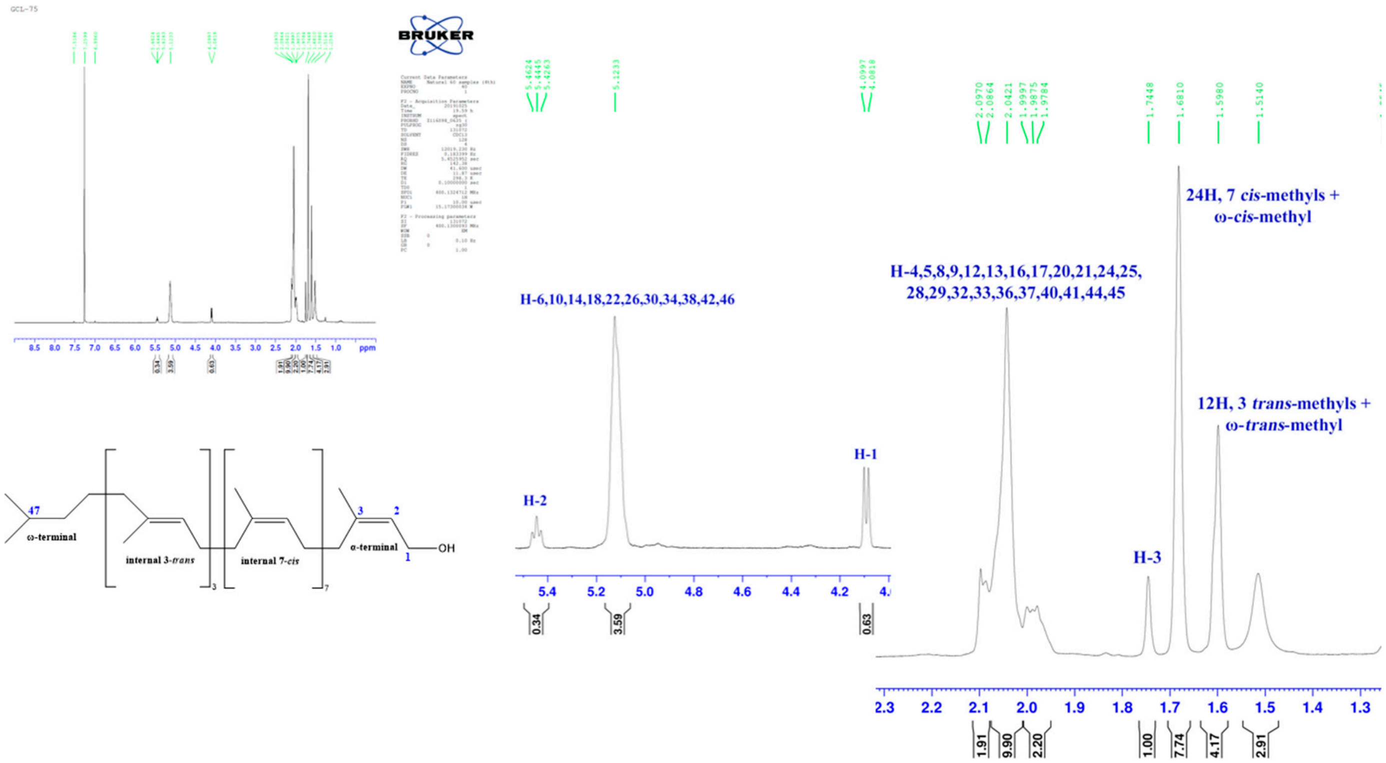

Figure S8. ^{1}H NMR spectrum (400 MHz, $CDCl_3$) of Compound 3.

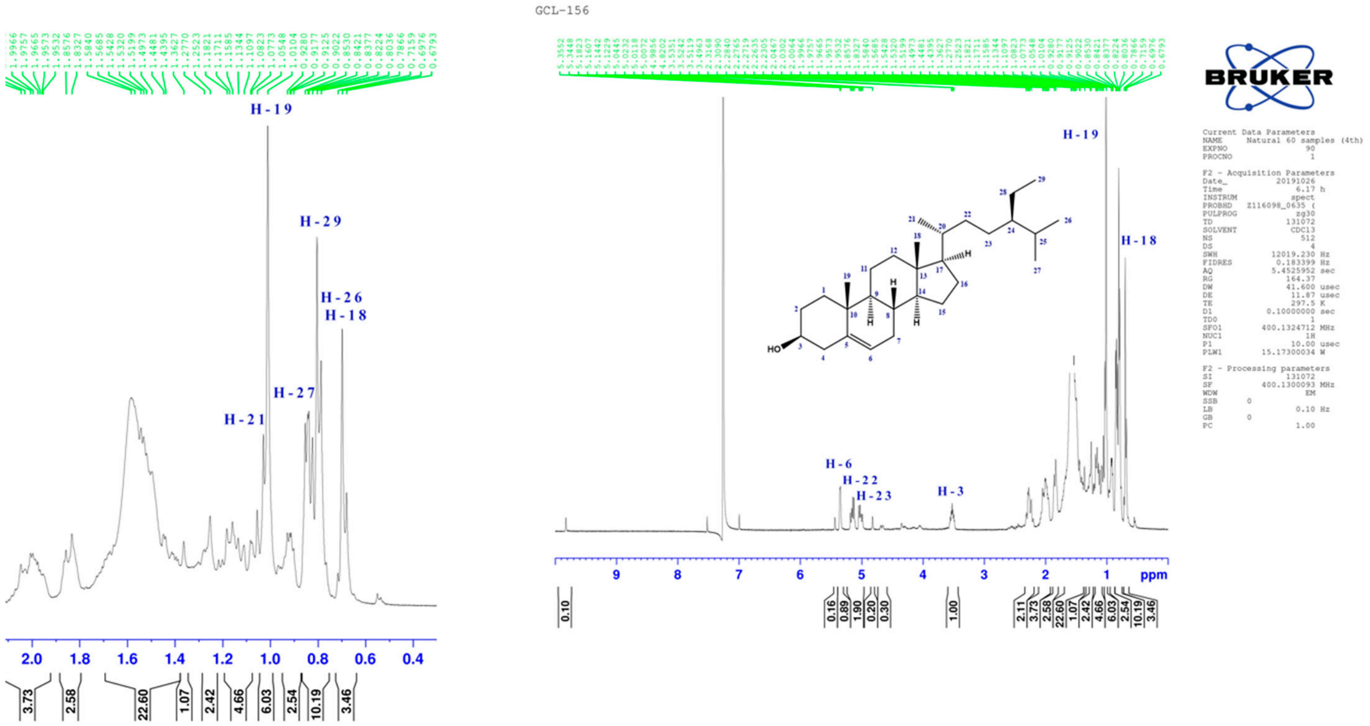

Figure S9. ^{1}H NMR spectrum (400 MHz, $CDCl_3$) of Compound 4.

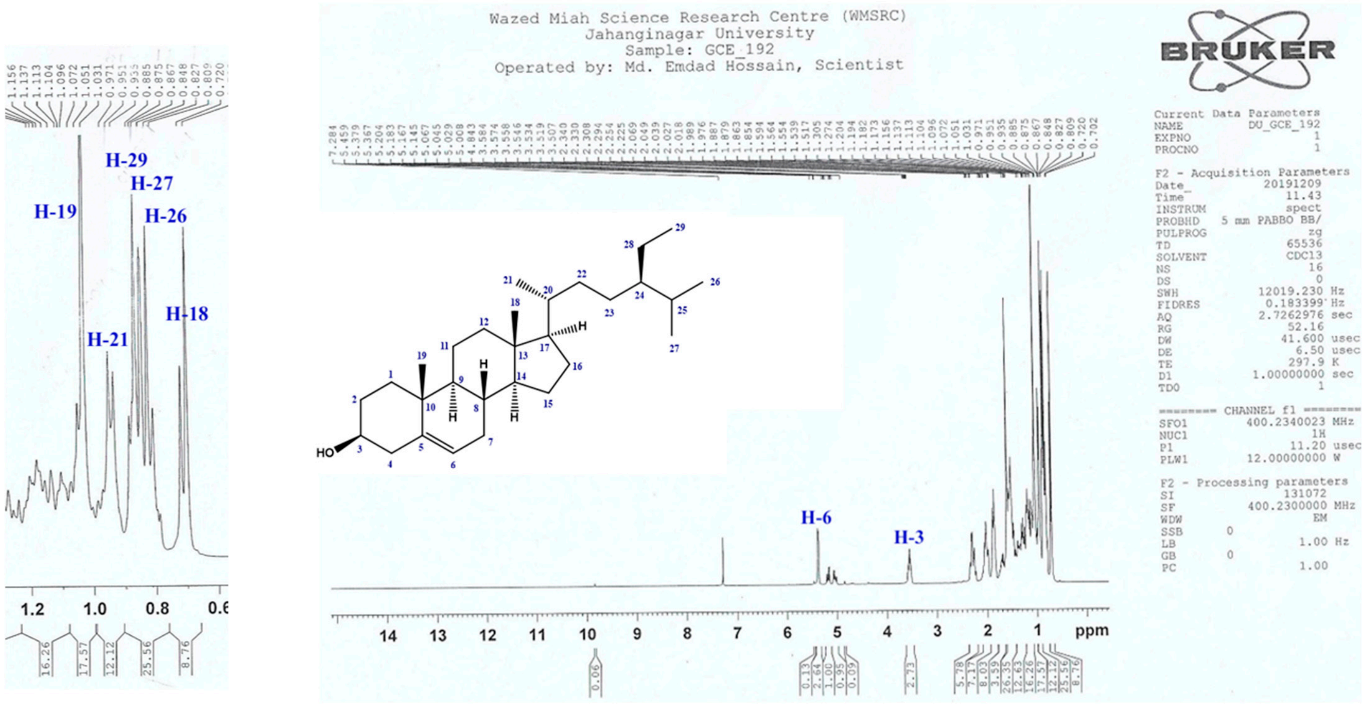

Figure S10. ^{1}H NMR spectrum (400 MHz, $CDCl_3$) of Compound 5.
